# Micro-CT Imaging of Pediatric Thyroglossal Duct Cysts: A Prospective Case Series

**DOI:** 10.3389/fped.2021.746010

**Published:** 2021-09-07

**Authors:** Claire Frauenfelder, Susan C. Shelmerdine, Ian C. Simcock, Andrew Hall, John Ciaran Hutchinson, Michael T. Ashworth, Owen J. Arthurs, Colin R. Butler

**Affiliations:** ^1^Great Ormond Street Hospital for Children NHS Foundation Trust, London, United Kingdom; ^2^Discipline of Surgery, School of Medicine, University of Adelaide, Adelaide, SA, Australia; ^3^UCL Great Ormond Street Institute of Child Health, Great Ormond Street Hospital for Children, London, United Kingdom; ^4^National Institute for Health Research Great Ormond Street Hospital Biomedical Research Centre, London, United Kingdom; ^5^Department of Histopathology, St Thomas' Hospital, London, United Kingdom

**Keywords:** diagnosis, radiology/imaging, thyroglossal duct cyst, head and neck, pathology

## Abstract

**Objectives:** To determine the feasibility of micro-CT as a high-resolution 3D imaging tool for thyroglossal duct cysts and to evaluate its role augmenting traditional histopathological examination of resected specimens.

**Methods:** A single centre, prospective case series of consecutive children undergoing excision of a thyroglossal duct cyst was performed at a quaternary paediatric referral hospital in the United Kingdom. Consecutive children listed for excision of a thyroglossal duct cyst whose parents agreed to participate were included and there were no exclusion criteria.

**Results:** Surgically excised thyroglossal duct cyst or remnant specimens from five patients (two males, three females) were examined using micro-CT alongside traditional histopathological examination. In all cases, micro-CT imaging was able to demonstrate 3D imaging datasets of the specimens successfully and direct radio-pathological comparisons were made (**Figures 1**–**5**, Supplementary Video 1).

**Conclusions:** The study has shown the feasibility and utility of post-operative micro-CT imaging of thyroglossal duct cysts specimens as a visual aid to traditional histopathological examination. It better informs the pathological specimen sectioning using multi-planar reconstruction and volume rendering tools without tissue destruction. In the complex, often arborised relationship between a thyroglossal duct cyst and the hyoid, micro-CT provides valuable image plane orientation and indicates proximity of the duct to the surgical margins. This is the first case series to explore the use of micro-CT imaging for pediatric thyroglossal duct specimens and it informs future work investigating the generalizability of micro-CT imaging methods for other lesions, particularly those from the head and neck region where precisely defining margins of excision may be challenging.

## Introduction

Thyroglossal duct cysts account for up to 70% of pediatric neck masses encountered in clinical practice (1, 2). A clear understanding of the embryological path of the thyroid gland and its relationship to the hyoid bone, tongue base musculature, and anterior neck tissue are key to successful surgery. As far back as 1893, Schlange described excision of the mid-portion of the hyoid bone during thyroglossal duct cyst surgery (3), while Sistrunk in his eponymous procedure for excision of thyroglossal duct cysts expanded the operation to include a block of midline tissue (4, 5). Removing the entirety of the remnant tract along the path of the primitive thyroid descending confers higher likelihood of complete excision of the lesion and significantly decreases recurrence, which occurs in up to 10% of patients following primary surgical excision and over 20% following revision procedures (6–8).

Histopathological assessment of the removed tissue is the current standard in confirming the diagnosis and complete surgical excision of the tract. One of the main drawbacks of this traditional approach is that sectioning is only possible in a single plane (typically longitudinal) as the tissue is destroyed during sectioning. In the past, imaging methods for reviewing surgical specimens were unable to provide the magnification and resolution required for detailed assessment, however novel strategies are now emerging.

Micro-focus computed tomography (micro-CT) imaging is being increasingly used as a non-destructive method for digital specimen analysis. It uses multiple X-rays to create a high-resolution 3D volume imaging dataset at a spatial resolution comparable to light microscopy (voxel size down to 1 micron) (9). At our institution, we currently provide a post-mortem fetal micro-CT clinical imaging service (10–12), however only a few publications have reported using this technology for excised human surgical specimens (13–16) and the utility of this imaging modality for pediatric thyroglossal duct cyst specimens is unknown, but clearly has appeal in providing high resolution 3D imaging of complex neck structures to aid pathological dissection.

The objectives of this study were two-fold: to determine whether micro-CT can provide high resolution imaging datasets to diagnose thyroglossal duct cysts and tracts, and, to evaluate the role for micro-CT imaging in aiding histopathological evaluation in this setting.

## Materials and Methods

### Setting and Participants

In this single center, prospective study we recruited consecutive children undergoing excision of a thyroglossal duct cyst over 11 months whose parents agreed to participate (5 November 2019–5 October 2020). Only those with written parental consent for micro-CT imaging of the post-operative specimen were included in the final study cohort. There were no exclusion criteria.

All patients underwent a routine extended Sistrunk's procedure (17). A midline neck skin incision was made with subplatysmal flaps elevated. A comprehensive block of midline neck tissue was excised, including infrahyoid tissue to the level of the thyroid isthmus, the medial adjacent strap muscles, the mid-portion of the hyoid bone, and the submucosal tongue base musculature superiorly. The cyst and any remnant thyroglossal duct tract are contained within the specimen block and may not be separately visualized if not active or previously ruptured during infection (or unsuccessful primary surgery). Where previous infection had ruptured through skin, the scarred overlying skin was excised as well.

### Tissue Preparation

All specimens were immersed in a solution of 10% formalin and potassium triiodide (I_2_KI; total iodine content of 63.25 mg/mL). Specimens were stored at room temperature for 72 h until fully iodinated then rinsed and dried before micro-CT imaging.

### Micro-Focus Computed Tomography Examination

Imaging of the specimens were acquired using 1 of 2 micro-CT scanners located on-site (XTH 225 ST or Med-X Alpha; Nikon Metrology, Tring, United Kingdom), both equipped with a multi-metal target. All imaging was undertaken by trained researchers (I.C.S or S.C.S). Specimens were secured within the scanner using foam supports, moisture absorbent wrapping material, and Parafilm M (Bemis Company, Inc., Oshkosh, WI) to ensure mechanical stability.

Imaging parameters varied according to specimen size, with X-ray energies and beam current ranging between 100 and 120 kV and 133 and 180 mA, respectively. Exposure times ranged from 354 to 500 ms, with 2 X-ray frames per projection and total number of projections varying between 2,301 and 3,141. Projection images acquired by the scanner were reconstructed using modified Feldkamp filtered back-projection algorithms with proprietary software (CTPro3D; Nikon Metrology, United Kingdom) and post-processed using VGStudio MAX 3.0 (Volume Graphics GmbH, Heidelberg, Germany). Isotropic voxel sizes varied according to specimen size and magnification, ranging from 8.18 to 22.19 microns.

### Histopathological Examination

After imaging samples were embedded in paraffin and processed by standard protocols, including staining with Haematoxylin and Eosin (H&E). The histopathological sections were reviewed by a pediatric pathologist (M.T.A) with >30 years of experience.

### Image Analysis

All micro-CT images were evaluated side by side with the histopathology results by two pediatric radiologists (O.J.A. and S.C.S.) with 4 years of micro-CT imaging reporting experience and a pediatric pathologist (M.T.A.). The radiologists and pathologist were not blinded to the clinical history. Images were evaluated to gain comparative radiological-pathological views, and contribution to diagnosis was discussed.

### Reporting

This case series has been reported in line with the PROCESS Guideline (18).

## Results

### Patient and Specimen Details

Over the 11-month study period, five patients who underwent primary excision of presumed thyroglossal duct cysts were recruited. The patient demographics, abbreviated clinical histories, and histopathological results are presented in Table 1. Lesions were excised from two male and three female patients aged 16 months to 14 years old.

**Table 1:** Patient demographics and clinical details.

**Case**	**Age/Gender**	**Primary/Previous infections**	**Clinical and procedural details**	**Specimen size (cm)**	**Outcome**
1	4 years, Female	Primary	18-month history of recurrent midline neck swelling. No previous infections or surgery. Extended Sistrunk's procedure performed.	3.0 × 0.6 × 1.0	Completely excised cystic tract, consistent with thyroglossal duct cyst. No atypia or malignancy.
2	5 years, Male	Primary	4-month history soft midline neck swelling. No previous infections or surgery. Extended Sistrunk's procedure performed.	3.5 × 2.5 × 1.5	Completely excised thyroglossal duct cyst. No atypia or malignancy.
3	6 years, Male	Previous infections with discharge through skin	Recurrent midline neck mass from 6 months of age. Multiple infections and discharge through skin. Overlying scarred skin excised during extended Sistrunk's procedure.	3.0 × 2.5 × 1.5	Completely excised thyroglossal duct cyst with overlying skin. No atypia or malignancy.
4	16 months, Female	Previous infections with discharge through skin	Recurrent midline neck swelling from 8 months of age. Multiple infections and discharge through skin. Overlying scarred skin excised during extended Sistrunk's procedure.	1.0 × 0.4 × 2.0	Completely excised thyroglossal duct remnant with overlying skin. No cyst identified. No atypia or malignancy.
5	14 months, Female	Previous infections with discharge through skin	6-year history of recurrent neck swelling right of midline with multiple infection. Small scar present. Overlying scarred skin excised during extended Sistrunk's procedure.	4.0 × 2.0 × 1.2	Completely excised thyroglossal duct remnant. No atypia or malignancy.

*Case number assigned in chronological order by date of operative procedure. Patient age denotes the age at time of the operation*.

In two cases, a primary extended Sistrunk's procedure was performed (i.e., no previous infection or rupture of the thyroglossal duct cyst, nor surgical intervention). In three cases, the surgery was performed due to recurrent cyst infections, which had led to discharge through the skin on at least one occasion prior to the surgery. For these cases, the overlying skin incorporating the scar was also excised in continuity with the routine operative specimen. Post-operative recovery for all patients was unremarkable. There was no evidence of recurrence in the 12 months following surgery.

### Radio-Pathological Correlation

**Figure 1:**
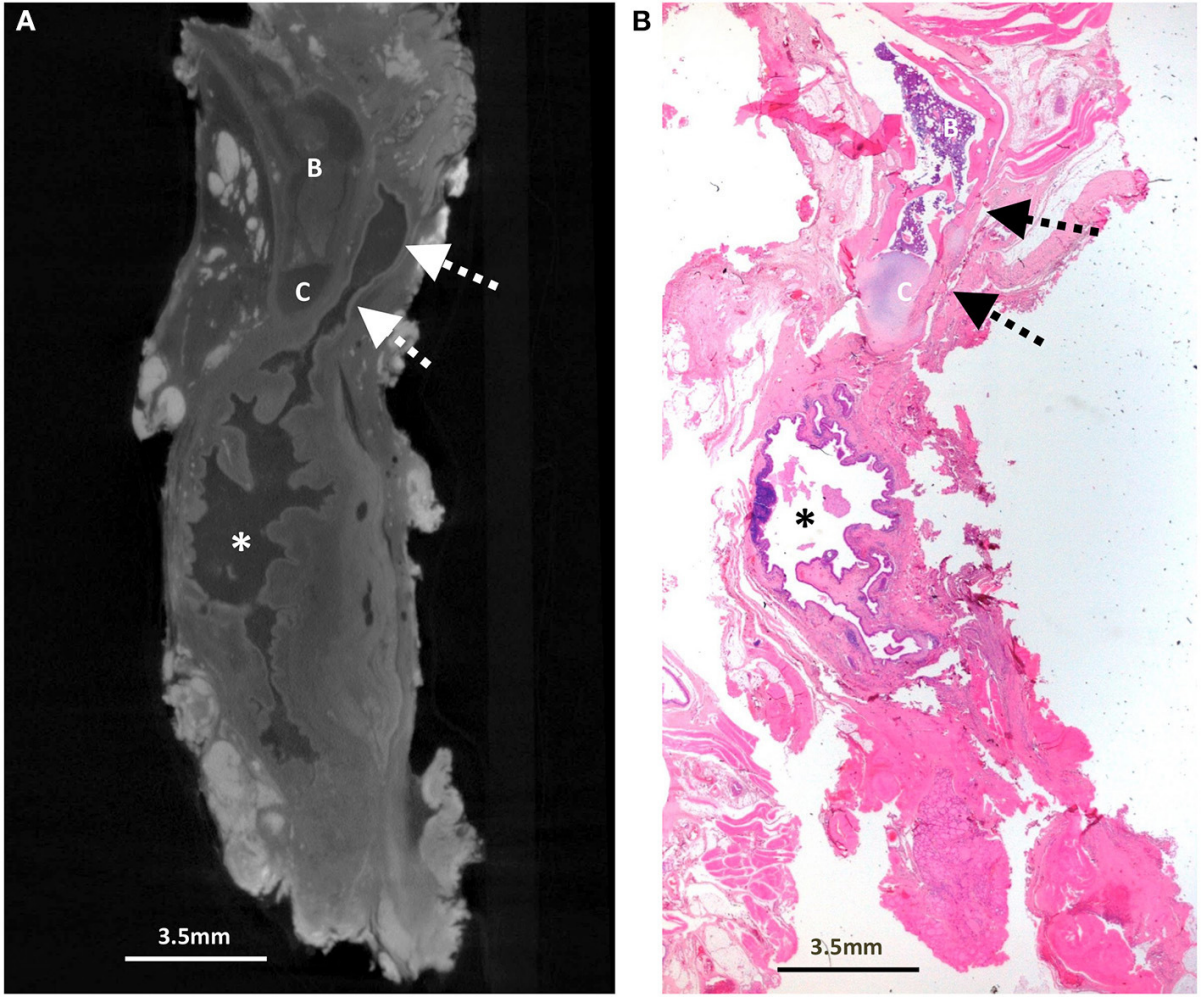
Excised thyroglossal duct cyst specimen from a 4-year-old female patient (case 1). **(A)** Paired iodinated micro-CT imaging at 17.3 micron resolution and **(B)** histopathological section with H&E staining in sagittal section demonstrate the thick-walled thyroglossal duct cyst (*) and the thyroglossal duct (dashed arrows) anterior to the hyoid bone (B) and hyoid cartilage (C).

**Figure 2:**
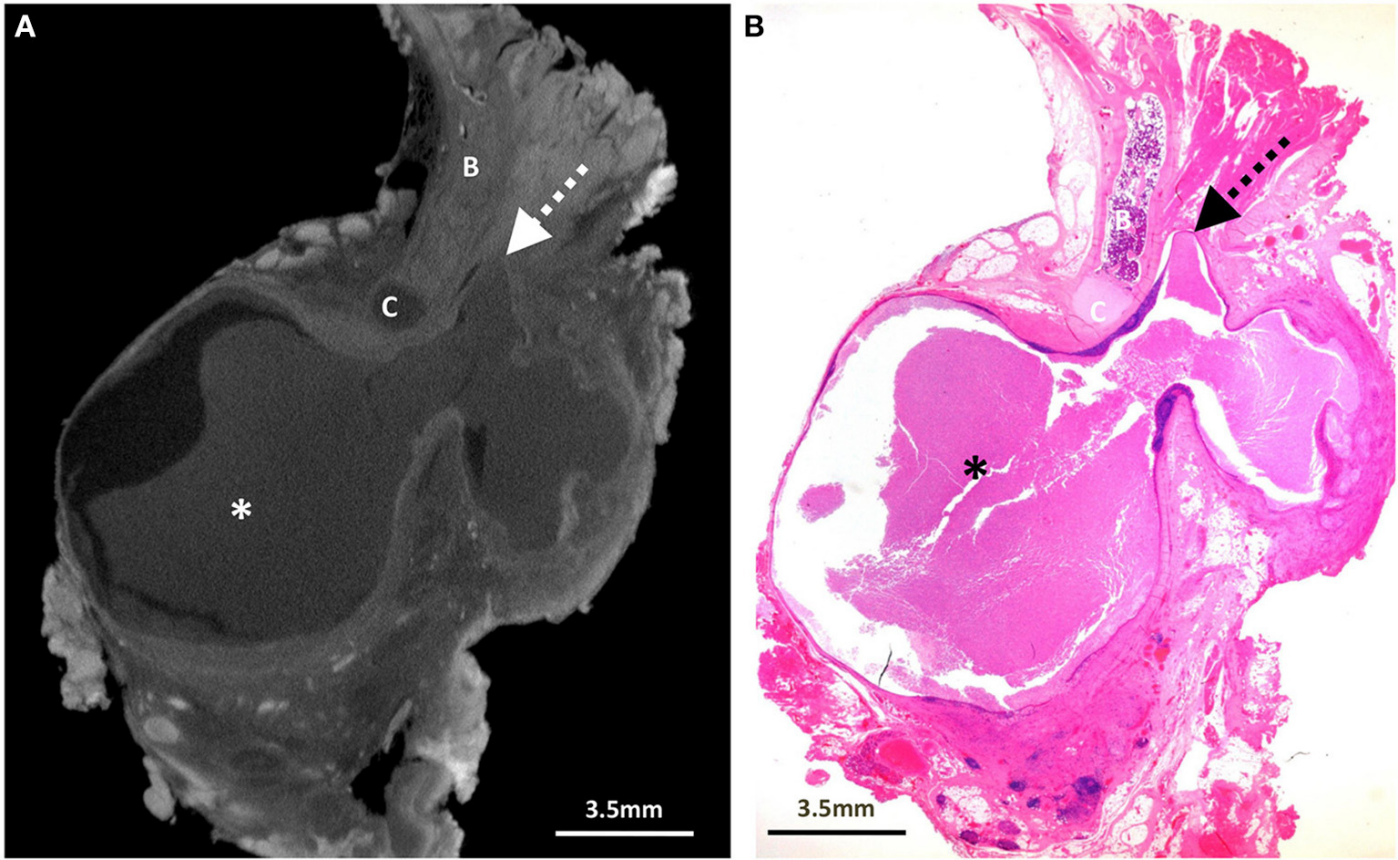
Excised thyroglossal duct cyst specimen from a 5-year-old male patient (case 2). **(A)** Paired iodinated micro-CT imaging at 20.0 micron resolution and **(B)** histopathological section with H&E staining in sagittal section demonstrate a large bilobed thyroglossal duct cyst (*) with mixed internal contents containing blood and pus, leading to a blind ending thyroglossal duct (dashed arrows) anterior to the hyoid bone (B) and hyoid cartilage (C).

**Figure 3:**
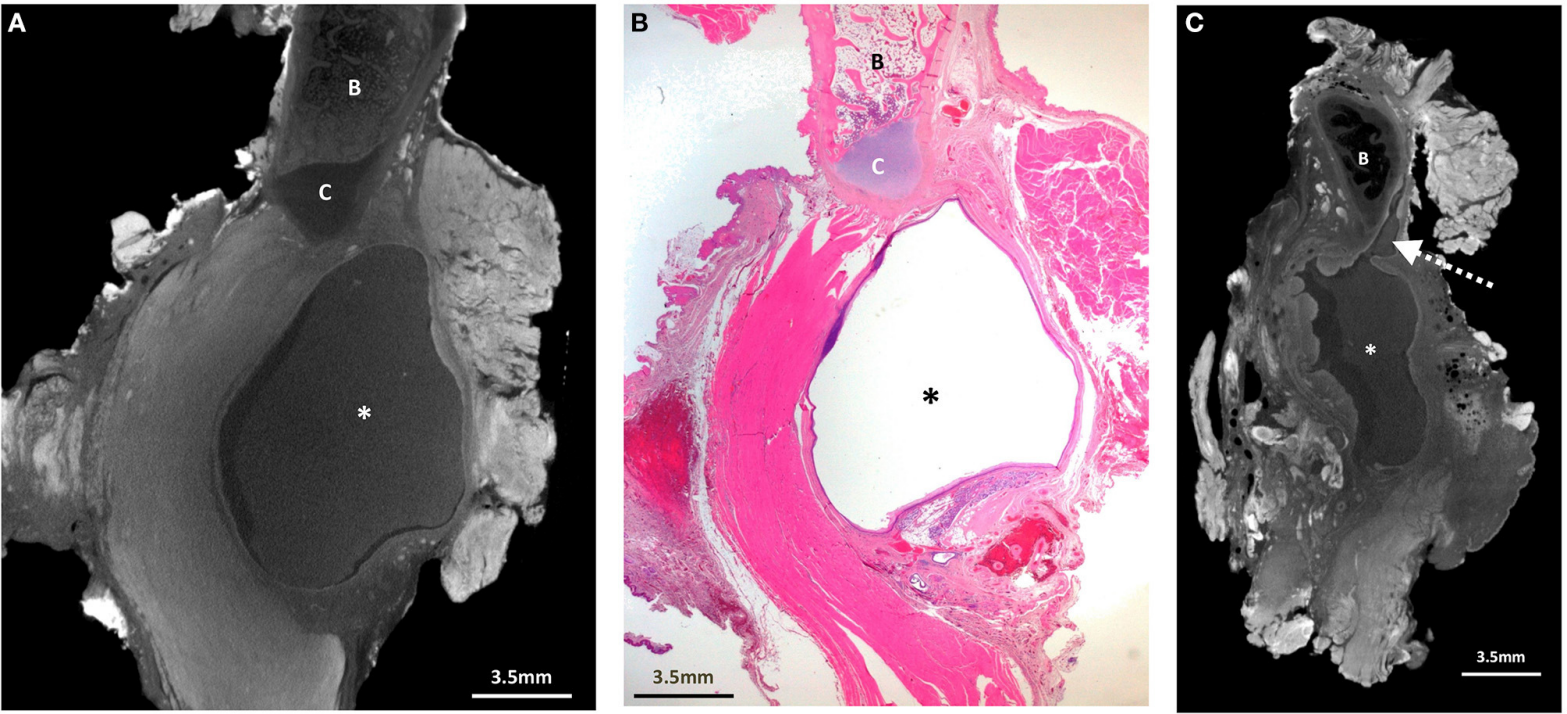
Excised thyroglossal duct cyst specimen from a 6-year-old male patient (case 3). **(A)** Paired iodinated micro-CT imaging at 19.9 micron resolution, and **(B)** histopathological section with H&E staining in sagittal section demonstrate a well encapsulated thyroglossal duct cyst (*), just anterior to the hyoid cartilage (C). The hyoid bone is demonstrated by the “B.” Although the thyroglossal duct was not present in the histopathological section, it was possible from the 3D imaging dataset to reconstruct the **(C)** sagittal viewing plane for better duct visualisation (dashed arrow).

**Figure 4:**
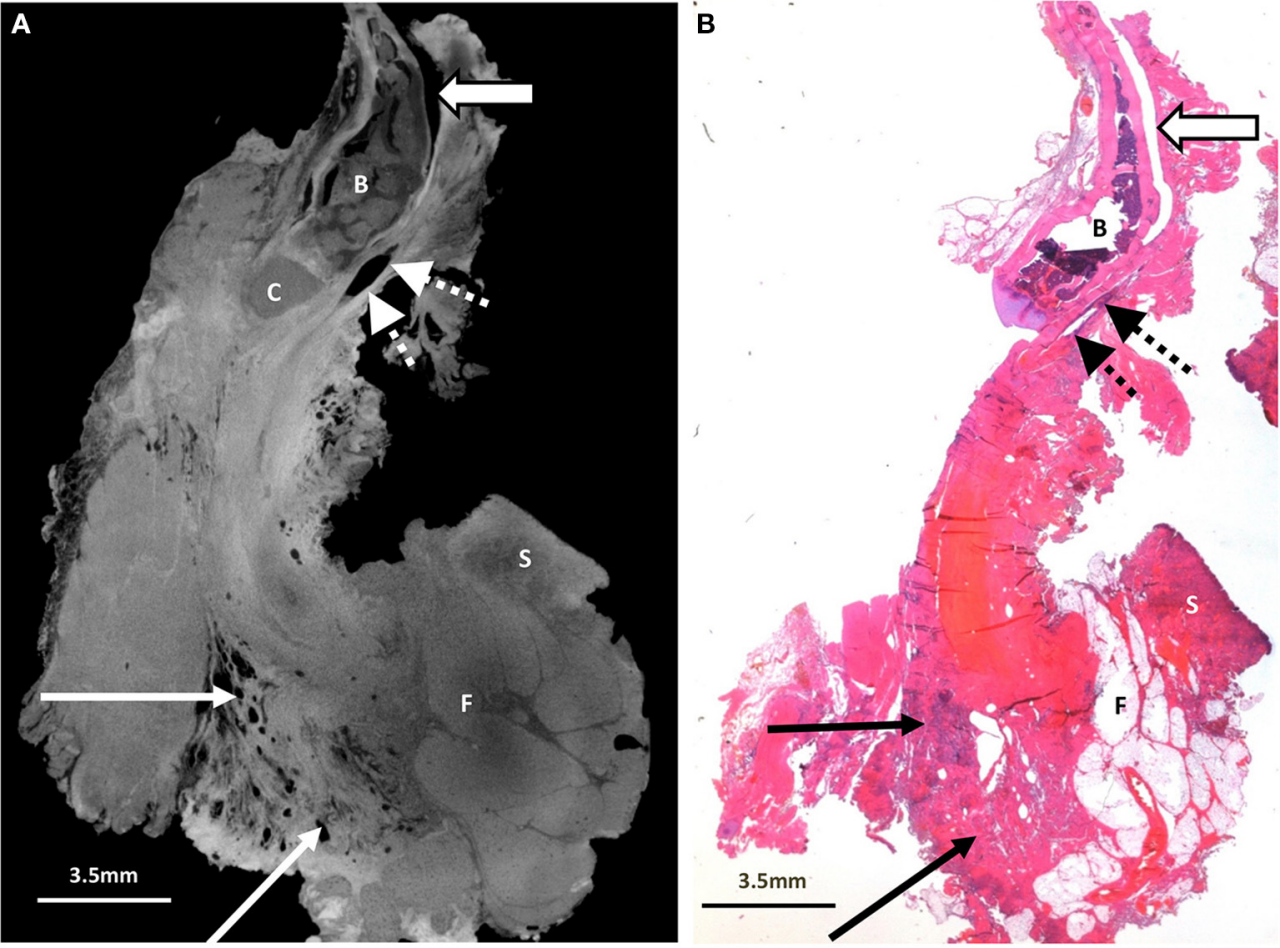
Excised thyroglossal duct cyst specimen from a 16-month old female patient (case 4). **(A)** Paired iodinated micro-CT imaging at 14.2 micron resolution and **(B)** histopathological section with H&E staining demonstrate the thyroglossal duct (dashed arrows) anterior to the hyoid bone (B) and hyoid cartilage (C). An artefactual cleft is demonstrated with the open arrow. There is no cystic structure on this image. Some skin (S) and fatty tissue (F) from the neck was removed at surgery, and areas of chronic inflammation with tethering of the adjacent musculature and surrounding tissues (solid arrows) are shown.

**Figure 5:**
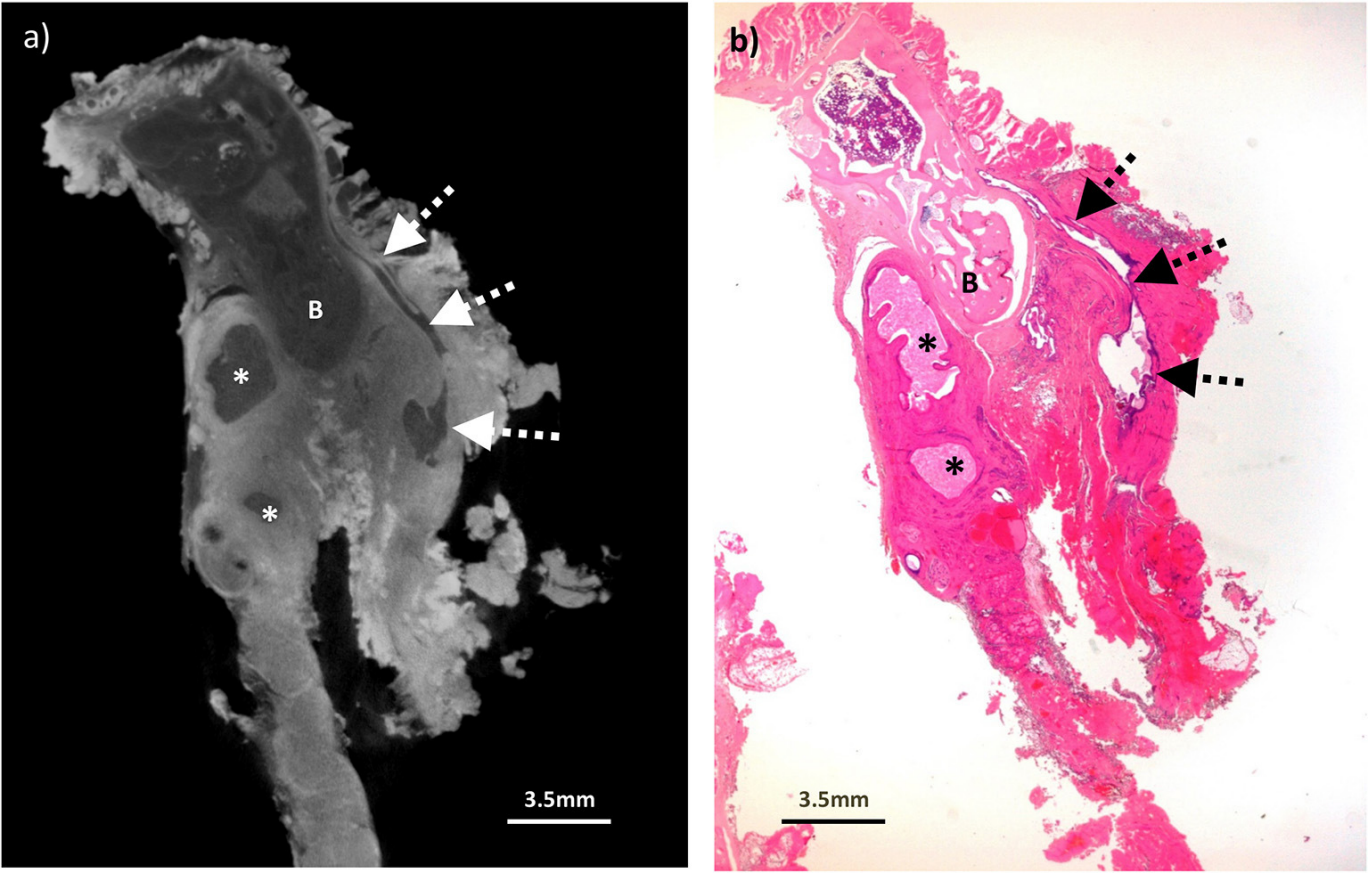
Excised thyroglossal duct cyst specimen from a 14-year old female patient (case 5). **(a)** Paired iodinated micro-CT imaging at 22.2 micron resolution and **(b)** histopathological section with H&E staining demonstrate the thyroglossal duct (dashed arrows) anterior to the hyoid bone (B) with areas of cystic change (remnants) from a previously infected and ruptured cyst (*).

In three cases, a thyroglossal duct cyst was identified, and in two cases a thyroglossal duct remnant (without cyst) was found. In all cases, micro-CT imaging was able to demonstrate 3D imaging datasets of the specimens, which were reformatted to create the same macroscopic appearances as histopathology on low magnification, light microscopy assessment. There were no additional structural features seen at histological assessment that were not visible on the micro-CT imaging. Direct radio-pathological comparisons between the micro-CT imaging and histopathological specimen appearances are provided in Figures 1–5, Supplementary Video 1. There were no cases of malignancy or cellular atypia in the specimens.

Surgeons were able to subjectively understand the Micro CT images more easily than the histopathology sections, and in combination they provided an excellent guide to interpreting complete surgical excision in these cases. Micro-CT also allowed for a 3D imaging “stack” to be stored in a digital format, which was particularly helpful for one of the cases (Figure 3, Supplementary Video 1) where the thyroglossal duct was not included in the pathological section but could be clearly delineated by reconstructing the micro-CT images.

Cellular appearances from high magnification, histopathological review (for ruling out cellular atypia or malignancy) were beyond the image resolution and magnification for the micro-CT imaging technology, however having a global overview of the specimen appearances allowed the pathologist to have a more informed approach when sectioning the specimens for subsequent histopathological assessment and for multidisciplinary discussions with the surgeons regarding completeness of the excision margins.

## Discussion

In this case series, we demonstrate the feasibility of micro-CT for imaging thyroglossal duct cyst specimens and reveal how high-resolution 3D imaging datasets can aid pathological dissection and demonstrate surgical excision, using histopathology results as a comparator/comparison.

The main advantages therefore conferred by using the micro-CT technique over traditional histopathological sectioning included the ability to review internal structures using multi-planar reconstruction and volume rendering tools without tissue destruction or sectioning. This was of particular relevance in this particular context because it helped to provide a detailed overview of the anatomical appearances of the thyroglossal duct, relationship to the hyoid bone and information on proximity of the duct to the surgical margins in a variety of planes. Post-excision recurrence is often attributed to the complex structure of the thyroglossal duct and incomplete excision of the arborised tract. Micro-CT enabled the pathologist to understand the optimal plane for tissue sectioning to display the key findings. The visualization of the micro-CT images, from a surgical perspective, were also subjectively easier to understand than those of the histopathology sections, and provided a useful adjunct in clinicians' interpretation of the cases.

To our knowledge, this is the first case series to explore the use of micro-CT imaging for pediatric thyroglossal duct specimens. Previous work has already discussed the benefits of this technique for other uses, such as for imaging excised whole organs [e.g., cardiac (19), brain (20)] as well as assessing cartilage rings in a pediatric case of tracheal stenosis (13). Micro-CT is becoming a more widely accepted tool for non-invasive post-mortem whole body fetal imaging (10, 12), as it allows internal visualization without surgical dissection. These studies have also demonstrated that iodine based micro-CT does not affect the histologic or immunohistochemical phenotype of normal tissues [skin biopsies and blood vessels (21) or diseased tissues, including cardiomyopathic cardiac tissue (22), cystic kidneys (23), and in neoplastic lesions of the heart (24), and brain (20)]. It has also been extensively used in mouse studies (25) and in human fetal autopsy (10) with subsequent histological examination, without reported alteration in subsequent tissue phenotype.

However, micro-CT availability is currently limited to specialist centres, and requires technological expertise for image acquisition and interpretation. It also cannot achieve imaging resolution to provide detailed cellular assessment, thus not replacing traditional histopathological assessment in its entirety. Through multi-disciplinary collaboration and comparison with histopathological sectioning, our centre has rapidly built experience in interpreting these radio-pathological appearances.

Micro-CT has the drawback of requiring exogenous contrast for surgical specimen imaging, necessitating a 72-h delay in our study. This was not an issue in this specific indication but could limit the usage of this technique where more urgent histopathology feedback is required. It is also currently unknown whether this iodination staining would affect more specialized pathology techniques (e.g., DNA extraction methods) downstream, if later required. For most thyroglossal duct cysts this may not be relevant but may impact the rare clinical situations where deposition of thyroid malignant tissue may be present within the excised cyst (26, 27). A recent study of micro-CT in thyroid cancer successfully evaluated capsular and vascular invasion and metastatic lymph node volume in specimens and was incorporated into pathology department workflow (28).

Limitations to our study include the small sample size and single centre design, although we have included a range of patient ages with varying clinical history including previously infected cysts, those which have been decompressed and primary surgical cases with an intact cyst. Pre-iodination contrast enhancement of our specimens could have caused a small amount of tissue shrinkage secondary to dehydration of the specimen [as previously reported (28)], and although we did not assess this in detail, no specimen degradation or significant impact on the histopathological diagnosis was found. Even at the highest possible micro-CT image magnification, microscopic cellular detail was not possible. Future studies could investigate whether micro-CT imaging could be undertaken to a similar effect and quality across a larger case load.

## Conclusion

In conclusion, we have shown the feasibility and utility of post-operative micro-CT imaging of thyroglossal duct cysts specimens, particularly in allowing for 3-D reconstruction in different planes without tissue destruction, and ability to store the specimen data in a digital format. It appears to be a helpful adjunct in specimen evaluation but does not currently replace histopathological assessment. Future work will focus on the generalizability of micro-CT imaging methods for other paediatric ENT specimens particularly those from the head and neck region where precisely defining margins of excision may be challenging (e.g., malignancies close to key structures) as well as investigating techniques to reduce time delays for tissue staining.

## Data Availability Statement

The raw data supporting the conclusions of this article will be made available by the authors, without undue reservation.

## Ethics Statement

The studies involving human participants were reviewed and approved by the National Health Service (NHS) Health Research Authority (HRA) (Research Ethics Committee Reference 17/WS/0089, awarded April 2017; IRAS ID: 222334). All samples were handled in accordance with the Human Tissue Act 2004. Written informed consent to participate in this study was provided by the participants' legal guardian/next of kin. Written informed consent was obtained from the minor(s)' legal guardian/next of kin for the publication of any potentially identifiable images or data included in this article.

## Author Contributions

CF, SS, AH, CB, and IS contributed to conception and design of the study. CF wrote the first draft of the manuscript. SS, AH, CB, IS, JH, MA, and OA wrote sections of the manuscript. All authors contributed to manuscript revision, read, and approved the submitted version.

## Funding

SS was supported by a RCUK/UKRI Innovation Fellowship and Medical Research Council (MRC) Clinical Research Training Fellowship (grant no. MR/R002118/1). This award was jointly funded by the Royal College of Radiologists (RCR). IS was funded by a National Institute for Health Research (NIHR) Clinical Doctoral Research Fellowship (ICA-CDRF-2017-03-53). OA was funded by an National Institute for Health Research (NIHR) Career Development Fellowship (NIHR-CDF-2017-10-037).

## Conflict of Interest

The authors declare that the research was conducted in the absence of any commercial or financial relationships that could be construed as a potential conflict of interest.

## Publisher's Note

All claims expressed in this article are solely those of the authors and do not necessarily represent those of their affiliated organizations, or those of the publisher, the editors and the reviewers. Any product that may be evaluated in this article, or claim that may be made by its manufacturer, is not guaranteed or endorsed by the publisher.

## Supplementary Material

Supplementary Video 1 Sagittal & Axial micro-CT images of excised thyroglossal duct cyst specimen in a 6-year-old patient. At histopathology section, the thyroglossal duct was not included, however in this video it is clearly shown anterior to the hyoid bone leading to the large cystic structure inferiorly. Please review in conjunction with Figure 3.
